# An information-oriented paradigm in evaluating accuracy and agreement in radiology

**DOI:** 10.1186/s41747-023-00327-y

**Published:** 2023-03-20

**Authors:** Alberto Casagrande, Francesco Fabris, Rossano Girometti

**Affiliations:** 1 Dipartimento di Matematica e Geoscienze, Università degli Studi di Trieste, Trieste, Italy; 2 Istituto di Radiologia, Dipartimento di Area Medica, Università degli Studi di Udine, Udine, Italy

**Keywords:** Diagnostic imaging, Information theory, Observer variation, Research design, Sensitivity and specificity

## Abstract

The goal of any radiological diagnostic process is to gain information about the patient’s status. However, the mathematical notion of information is usually not adopted to measure the performance of a diagnostic test or the agreement among readers in providing a certain diagnosis. Indeed, commonly used metrics for assessing diagnostic accuracy (*e.g.,* sensitivity and specificity) or inter-reader agreement (Cohen $\kappa$ statistics) use confusion matrices containing the number of true- and false positives/negatives results of a test, or the number of concordant/discordant categorizations, respectively, thus lacking proper information content. We present a methodological paradigm, based on Shannon’s information theory, aiming to measure both accuracy and agreement in diagnostic radiology. This approach models the information flow as a “diagnostic channel” connecting the state of the patient’s disease and the radiologist or, in the case of agreement analysis, as an “agreement channel” linking two or more radiologists evaluating the same set of images. For both cases, we proposed some measures, derived from Shannon’s mutual information, which can represent an alternative way to express diagnostic accuracy and agreement in radiology.

**Key points**

• Diagnostic processes can be modeled with information theory (IT).

• IT metrics of diagnostic accuracy are independent from disease prevalence.

• IT metrics of inter-reader agreements can overcome Cohen κ pitfalls.

## Background

One can hypothesize that any examination aims to extract as much information as possible about a disease (its presence and/or its gravity or stage), which can be assumed a “hidden,” objective status of a patient according to a standard of reference. Analogously, gauging the agreement in radiology can be seen as measuring how much information is shared between different readers assessing a certain condition, *e.g.,* by using the prostate imaging reporting and data system (PI-RADS) categories for assessing prostate cancer [1]. Of note, the notion of information, rather than being vague, can be expressed quantitatively and rigorously in accordance with the mathematical apparatus underlying the so-called information theory (IT), which is the base of current telecommunication systems technology [2]. Details on the mathematical definition of information and derived measures can be found in the milestone paper with which Claude Shannon founded IT in 1948 [3]. Based on the above assumptions, some IT-derived measures of both diagnostic accuracy and agreement have previously been built [4–7], proving their formal consistency. The purpose of this article is to present them to the radiological community, describing their conceptual fundaments and potential advantages compared to conventional statistics such as receiver operating characteristic (ROC) analysis and Cohen $\kappa$.

## The information-oriented paradigm

The mutual information (MI) [3] is a measure of the (average) quantity of information exchanged between a sender and a receiver communicating on a channel. It is defined by the formula as follows:

$$MI\left(X,Y\right)=\sum_{x\in \mathcal{X}}\sum_{y\in \mathcal{Y}}{p}_{XY}\left(x,y\right)*\mathrm{log}\frac{{p}_{XY}\left(x,y\right)}{{p}_{X}\left(x\right)*{p}_{Y}\left(y\right)}$$

where $X$ and $Y$ are two random variables modeling the input and the output of the channel, $\mathcal{X}$ and $\mathcal{Y}$ are the sets of the possible input and output messages, and ${p}_{X}(x)$, ${p}_{Y}(y)$, and ${p}_{XY}(x,y)$ denote the probability for *X* to equal *x*, the probability for *Y* to equal *y*, and the joint probability for *X* and *Y* to equal *x* and *y* at the same time, respectively.

MI measures the dependency between $X$ and $Y$: the more they are related, the higher the mutual information. Whenever the two variables are independent, *i.e.*, their values are non-related, MI drops to 0. The mutual information is symmetric and the role of the channel input and output is not relevant in gauging the information exchanged on the channel itself, *i.e.,* $MI\left(X,Y\right)=MI\left(Y,X\right).$

Because of its features, MI can also be used as a correlation index between $X$ and $Y$, but differently from the Pearson correlation coefficient that exclusively handles linear correlations, MI is a nonlinear index.

### Modeling diagnostic information with *MI*

The diagnostic channel $D\iff X$ is a virtual channel modeling the information flow between the unknown patient condition $D$ (*i.e.,* the channel input) and the outcome $X$ of a diagnostic test (*i.e.,* the channel output) [4, 6–8]. The diagnostic test relates the two random variables $D$ and $X$ as channels connect their input and output, and its goal is to carry the maximum amount of information of the former to the latter.

Since MI is symmetric, the diagnostic channel can be represented as symmetric too: the information flows from the patient condition to the test outcome exactly as it flows from the test outcome to the patient condition. Thus, if the diagnostic channels $D\iff X$ and $D\iff Y$ model two different diagnostic tests or readers, we can relate the outcomes $X$ and $Y$ of the two tests/readers by joining $D\iff X$ and $D\iff Y$ in the single channel $X\iff D\iff Y$, or, in short, $X\iff Y$. This last channel is the agreement channel [5–7].

The mutual information can be used to both evaluate the accuracy of the test modeled by a diagnostic channel $D\iff X$ and measure the agreement of two tests/readers linked by an agreement channel $X\iff Y$.

## An information measure of diagnostic accuracy

Image analysis can be seen as a way to extract information from the patient to correctly diagnose the disease. The most accurate diagnosis will be, in turn, the one extracting as much information as possible. Consequently, the more information flows on the diagnostic channel from the disease to the radiologist, the more accurate the diagnosis is.

### The dichotomous case

As shown in [4], whenever the evaluation has only two possible outcomes (dichotomous case), the amount of information flowing in a diagnostic channel, *i.e.,* MI, can be expressed in terms of sensitivity ($\mathrm{SE}$), specificity ($\mathrm{SP}$), and prevalence of the disease $\mathrm{PREV}$ as follows:

$$MI(SE, SP, PREV)= h\left(SP-\left(SE+SP-1\right)*PREV\right)+\left(h\left(SP\right)-h\left(SE\right)\right)*PREV-h\left(SP\right)$$

where $h\left(x\right)=-x*{\mathrm{log}}_{2}x-\left(1-x\right)*{\mathrm{log}}_{2}(1-x)$ is the (binary) *Shannon entropy* [3]. 

The IT-based approach avoids the dependency from the prevalence of the disease by assessing diagnostic accuracy in terms of the area under the curve (AUC) subtended by the *MI*-curve obtained by varying the prevalence for all possible values in the interval $\left[0,1\right]$, *i.e.,* a prevalence of disease ranging from $[0]$ to $100\mathrm{\%}$.

**Fig. 1:**
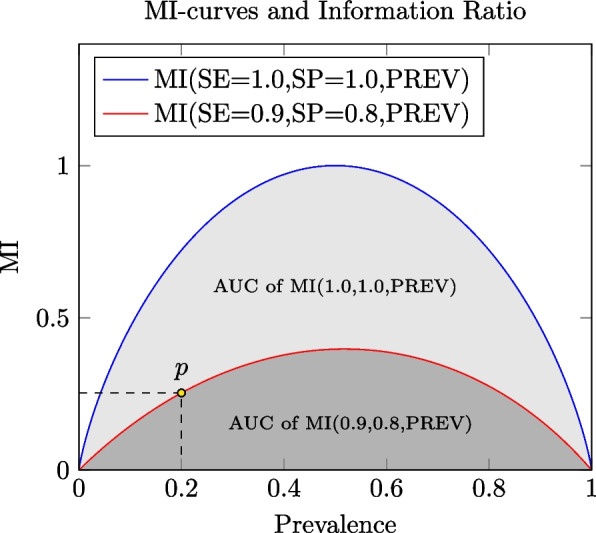
The red line is the MI-curve for SE 0.9 and SP 0.8 as the prevalence of the disease ranges in the interval [0, 1]. The point *p* represents the value of MI (value on the MI axis) when the prevalence is 0.2 (value on the prevalence axis). The dark gray region is the area under the MI-curve for SE 0.9 and SP 0.8. It summarizes all the values of MI as the prevalence changes for a diagnostic method whose sensitivity and specificity are 0.9 and 0.8, respectively. This AUC does not range in an interval [0, 1], and it never reach 1. In order to normalize it in the [0, 1] range and get the information ratio, we must divide it by the maximum of the AUC among those obtainable by changing the sensitivity and the specificity. This is the AUC of the diagnostic method that has the best sensitivity and specificity, *i.e.,* 1 and 1. The blue line depicts the MI-curve for SE 1.0 and SP 1.0, *i.e.,* the gold standard, and the gray region (*i.e.,* the light gray plus the dark gray regions) represents its AUC. In [4] it is proved that the latter equals $1/\mathrm{ln}4$; hence, the information ratio of the method whose SE and SP are 0.9 and 0.8, respectively, is the AUC of the red curve multiplied by $\mathrm{ln}4$. *AUC* Area under the curve, *MI* Mutual information, *SE* Sensitivity, *SP* Specificity, *PREV* Prevalence

The information ratio (IR) [4] is an information measure that normalizes MI-curve AUC with respect to the best possible performance. In the dichotomic case, IR can be computed by the formula

$$IR(SE,SP)=(\mathrm{ln}4)*{\int }_{0}^{1}MI\left(SE,SP,PREV\right)dPREV=1+\frac{\left(1-SP\right)*h\left(1-SE\right)+\left(1-SE\right)*h\left(1-SP\right)}{SE+SP-1}*\mathrm{ln}2+\frac{\left(1-SE\right)*\mathrm{ln}\left(1-SE\right)+\left(1-SP\right)*\mathrm{ln}\left(1-SP\right)}{SE+SP-1}$$

where $h\left(x\right)$ is the binary Shannon entropy as above. Figure 1 depicts two exemplificative MI-curves: MI for SE 0.9 and SP 0.8 as the prevalence changes and MI for the reference standard, *i.e.,* SE and SP equal to 1. The ratio between the AUC of the former and that of the AUC of the latter is the $\mathrm{IR}(0.9,0.8)$.

IR represents the normalized amount of information carried by a diagnostic process as a value in the interval $\left[0,1\right]$: the higher diagnostic accuracy, the nearer IR to 1.

Table 1 reports a simulated clinical scenario in which magnetic resonance imaging is used to identify clinically significant prostate cancer in a population of biopsy-naïve men with 40% cancer prevalence [9].

**Table 1:** Simulated clinical scenario in which magnetic resonance imaging is used to identify clinically significant prostate cancer in a population of 1000 biopsy-naïve men with 40% cancer prevalence

	PI-RADS category	Target biopsy	
		Positive	Negative
**Magnetic resonance imaging**	5	93	37
	4	201	128
	3	86	135
	2	18	131
	1	2	169
	*Total number of subjects*	*400*	*600*

By setting the cutoff for auctioning prostate biopsy to prostate imaging reporting and data system (PI-RADS) category 3, we get Table 2. Since the SE and SP of the data in this table are 0.95 (95%) and 0.5 (50%), respectively, $\mathrm{IR}(0.95,0.5)\approx0.195$. Hence, due to the imbalance between SE and SP (a typical state-of-the-art clinical scenario in this field), MRI can extract only 19.5% of the possible information about a patient prostate cancer condition on average.

**Table 2:** Simulated PI-RADS diagnoses by cut-off selection

		Target biopsy		
		Positive	Negative	
**Magnetic resonance imaging**	**Positive** (PI-RADS category ≥ 3)	380	300	*Positive predictive value 55.8% (380/680)*
	**Negative** (PI-RADS category ≤ 2)	20	300	*Negative predictive value 93.7% (300/320)*
		*Sensitivity 95% (380/400)*	*Specificity 50% (300/600)*	
		*Cohen κ*	*0.403*	
		*Information ratio*	*0.195*	

By arbitrary setting the cutoff to PI-RADS category 3 (*i.e.,* categories below 3 are considered a negative response, categories above 2 correspond to a positive response), we obtain Table 2, showing a sensitivity of 0.95 (95%) and a specificity of 0.50 (50%). It follows that the information ratio of the scenario is about 0.195, while for the same data Cohen κ equals 0.403

### The prevalence issue

While the IR value depends on the SE and SP values, as shown in the formula, IR is calculated by integrating them over all possible prevalence values included between 0 and 1; thus, in fact making the metric independent from it [4]. Therefore, the results expressed by using it are also valid in cohorts different from the one in which a study has been performed, *e.g.,* cohorts with different prevalences of the disease. Note, however, that many studies have suggested that both SE and SP are related to the prevalence itself [10–13]. This is a problem of spectrum bias, which is a type of sampling bias. In these cases, sampling from a patient population with a higher disease prevalence may include more severely diseased patients making the test performing better. Our analysis does not account for the effect of bias in study design like the abovementioned one. We are currently working on mathematical solutions for trying to overcome this problem.

### The multivalue case

**Fig. 2:**
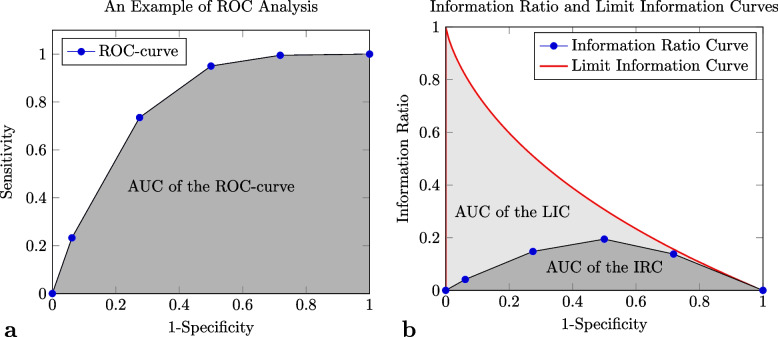
Receiver operating characteristic (ROC) and global information ratio (GIR) analyses of data reported in Table 1. The blue points in the figures denotes the 6 possible cutoffs, *i.e.,* all the category below the *i*th one, where $i\in\;\left[0,5\right]$ are considered negative diagnoses. **a** The ROC analysis plots the cutoff points in the (1—SP) × SE space, and it connects them by using the ROC curve. The area under this curve is a cutoff-independent measure of the effectiveness of the diagnostic approach: the higher the area, the better the approach. The ROC area under the curve (AUC) ranges in the [0, 1] interval. The figure represents the ROC curve and its AUC as a black line and a dark gray region, respectively. In the depicted scenario, the AUC is about 0.793. **b** The GIR analysis of the same data depicts the cutoff points in the (1—SP) × IR space. The point themselves are connected by the information ratio curve (IRC) which is represented as a black line. As the ROC AUC, the IRC AUC (the dark gray region in the figure) is a measure of the effectiveness of the diagnostic approach, but, since it is computed by using IR, it is prevalence-independent. Unfortunately, it does not range in the interval [0, 1], and to normalize it, it must be divided by the IRC AUC of the best theoretical diagnostic approach, *i.e.,* those whose sensitivity is always 1: the limit information curve (LIC). The *LIC* AUC (the light and dark gray regions in the figure) always equals $2-{\pi }^{2}/6$ [4]; thus, the ratio between IRC and LIC AUCs, *i.e.,* the global information ratio (GIR), equals IRC AUC divided by $2-{\pi }^{2}/6$. In the scenario depicted by panel **b**, IRC AUC and GIR are about 0.116 and 0.326, respectively

Radiologists frequently provide diagnoses by using ranks (*e.g.,* the PI-RADS) or measuring continuous variables (*e.g.*, the apparent diffusion coefficient from diffusion-weighted imaging [14]). In these cases, the receiver operating characteristics (ROC) analysis is commonly used to discover the most appropriate cutoff for the investigated condition identification and assess the diagnostic approach effectiveness. ROC analysis uses each of the values in the considered rank or continuous domain as a possible cutoff for a curated set of diagnoses, and it builds a 2 × 2 confusion matrix which relates the golden standard and the positive/negative results due to the specific cutoff. SE and SP of each of these matrices depend on the corresponding cutoff value: the greater the cutoff value, the smaller the sensitivity, and the greater the specificity and vice-versa, depending on the clinical scenario. By representing the possible cutoffs as points in a (1–SP) *versus* SP graph, ROC analysis produces a curve that depicts the effectiveness of the diagnostic method as the cutoff changes (Fig. 2a). The effectiveness is quantified by the AUC of the curve itself which is proven to be the probability for the rank/value assigned to a subject not having the investigated condition by the diagnostic method to be lower than that of a subject having the condition [15].

In a similar way, the IT-based approach evaluates the IRs of the cutoff-specific matrices, and it plots the information ratio curve (IRC), *i.e.,* the curve of IR as the cutoff is raised or, equivalently, as the specificity decreases. The IRC AUC does not range in the interval [0, 1]. In order to normalize it, it must be divided by the AUC of the limit information curve (LIC) that is the curve produced by the best theoretical diagnostic method: the one that always has the maximum sensitivity (*i.e.,* 1) for any admissible value of the specificity (Fig. 2b). This ratio is the global information ratio (GIR). Since LIC AUC always equals $2-{\pi }^{2}/6\approx 0.355$ [4], GIR can be computed as follows:

$$GIR=\frac{AU{C}_{_{IRC}}}{2-{\pi }^{2}/6}$$

### Advantages of the IT-based model for diagnostic accuracy

IR and GIR are guaranteed to be global, normalized, and prevalence-independent measures for diagnostic accuracy. The prevalence-independency is a certain advantage with respect to the standard Cohen κ and ROC analysis. Another issue related to the ROC analysis is the arbitrary choice of the optimal cutoff, which, even if reasonable (as in the case of the Youden index [16]), is not supported by any motivation related to the aim of any diagnostic process, which is to extract as much information as possible from the test. The GIR analysis is the only one to offer such a criterion, based on the maximization of the information flow between the patient and the clinician, which is the scope of any good diagnostic system.

## An information measure of inter-reader agreement

As already noticed above, the agreement channel $X\iff Y$ relates the outcomes of two diagnostic tests or readers. Hence, the mutual information over this channel, *i.e.,* $\mathrm{MI}\left(X,Y\right)$, can be used as a correlation index between the outcomes of the two readers, *i.e.,* $X$ and $Y$. Since $\mathrm{MI}(X,Y)\leq\min\{H\left(X\right),H\left(Y\right)\}$ [3, 5] where $H\left(X\right)$ and $H\left(Y\right)$ are the entropy associated with the variables $X$ and $\mathrm{Y}$, respectively, we can normalize $\mathrm{MI}(X,Y)$ in the interval $[\mathrm{0,1}]$ by dividing it by its maximum value, *i.e.,* $\mathrm{min}\{H\left(X\right),H\left(Y\right)\}$. This leads to the information agreement (IA):

$$IA\left(X,Y\right)=\frac{MI(X,Y)}{\mathrm{min}\{H\left(X\right),H(Y)\}}$$

whose value ranges in the interval $\left[0, 1\right]$. As pointed out in [5], IA provides an exact and coherent measure of the stochastic distance between ${P}_{XY}$ and ${P}_{X}{P}_{Y}$., that is the joint distribution and the product of the marginals. One might argue that such a determination is measured at a less formally rigorous and precise extent when using Cohen $\kappa$ [17]. However, IA can be thought of as a (normalized) measure of the degree of dependence between two readers when making a diagnosis. The greater the IA value, the higher the inter-reader agreement, *i.e.,* the inter-dependence of the readers. Of note, the measure can be used in both the dichotomous and multi-valued scale ratings [5], while it does not currently apply to continuous variables.

### Advantages of the IT-based model for inter-reader agreement

IA overcomes some of the well-known Cohen κ flaws [18–20]. This is partially testified by the three artificial inter-reader agreements presented in Table 3.

**Table 3:** Agreement matrices leading to paradoxical values for Cohen κ

		Reader 1	
		Positive	Negative
**Reader 2**	**Positive**	7,210	5,200
	**Negative**	120	7,470
		*Cohen κ*	*0.5*
		*Information agreement*	*0.311*

Tables 3, 4, and 5 compare three pairs of readers on the bases of 20,000 diagnoses. Since the readers considered in Table 3 agree 73.4% of the time, while 99.4% of the diagnoses coincide in Table 4, the latter scenario seems to deserve an agreement value greater than that presented in Table 3. However, both the tables have the very same Cohen κ, *i.e.,* 0.5. IA better fits common sense in this case because Tables 3 and 4 IAs equal 0.311 and 0.638, respectively.

**Table 4:** Agreement matrices leading to paradoxical values for Cohen κ

		Reader 1	
		Positive	Negative
**Reader 2**	**Positive**	19,818	116
	**Negative**	5	61
		*Cohen κ*	*0.5*
		*Information agreement*	*0.638*

**Table 5:** Agreement matrices leading to paradoxical values for Cohen κ

		Reader 1	
		Positive	Negative
**Reader 2**	**Positive**	19,818	156
	**Negative**	1	25
		*Cohen κ*	*0.240*
		*Information agreement*	*0.580*

Tables 3 and 4 have the same Cohen κ (*i.e.,* 0.5), but the number of accordant diagnoses in the former (*i.e.,* 73.4%) is significantly lower than those of the latter (*i.e.,* 99.4%). Information agreement properly measures this difference and assigns the values 0.311 and 0.638 to Tables 3 and 4, respectively. On the contrary, Tables 4 and 5 report quite similar scenarios, as they differ for less than 1% of the total diagnoses. However, the Cohen κ of the former (0.5) is much higher than that of the latter (0.240) whereas their information agreement (0.638 and 0.58, respectively) do not significantly deviate. In all these cases, the information agreement exhibits a behavior more consistent to the common sense of agreement than Cohen κ

Tables 4 and 5 are almost identical: they differ for less than 1% of the diagnoses. Because of this similarity, one may expect that their agreement values are almost the same. Still, their Cohen κ is quite different: 0.5 for Table 4 and 0.24 for Table 5. On the contrary, their IAs are 0.638 and 0.580, respectively. Once more, IA offers a more convincing agreement measure with respect to Cohen κ.

## Conclusions

IT is a rigorous mathematical tool widely used in electronic telecommunication systems. We presented some IT-inspired diagnostic accuracy and inter-reader agreement measures. While the underlying conceptual framework of these measures may be harder to be understood compared to currently used statistical indexes, it brings some appealing advantages in the presentation and interpretation of radiological research, such as ($i$) providing summary measures of accuracy not depending on the prevalence of disease, and ($ii$) assessing diagnostic accuracy and inter-reader agreement without potential pitfalls of conventional analysis.

On this basis, we suggest that the above-presented information-based indexes of diagnostic accuracy could complement conventional ones by objectively showing how much information on the patient status is truly captured by a diagnostic tool, given a definite set of SE and SP. This knowledge could be useful to assess whether any refined diagnostic strategy using that tool or new ones truly translates into a gain in information on the disease or compare the amount of information provided by different tools. This is potentially relevant in some settings such as testing artificial intelligence-based tools, which are supposed to extract additional information from medical images. Additional information could be quantified more precisely compared to conventional qualitative images or when comparing various algorithms.

Concerning agreement analysis, the index we proposed could be used as an alternative to Cohen κ as it is not affected by the abovementioned biases related to unbalanced data distribution in source 2 × 2 tables.

However, some points should be faced before information measures can be fully appreciated and used. First of all, easy-to-use software tools for evaluating them are missing. We are currently working on an online platform designed to obtain the accuracy or agreement information measures by entering data directly from a database and hope this can be readily available. Second, reference values for IR, GIR, and IA are not yet defined, thus making it difficult to establish which values can be qualified as “high or low accuracy” or rather can express different levels of agreement (*e.g.,* low, moderate, substantial, excellent), and in turn, limiting potential concrete applications. Furthermore, the rules for comparing different GIR values have not been established. Those limitations suggest that some additional mathematical work is needed to refine information indexes. Finally, a direct comparison of those measures with conventional ones in real study cohorts will be indispensable to understand how much informative and impacting the information indexes are compared to conventional ones, in the light of the potential advantages we described and make radiologists familiar with new indexes.

## Data Availability

Not applicable.

## Abbreviations

AUC: Area under the curve
GIR: Global information ratio
IA: Information agreement
IR: Information ratio
IRC: Information ratio curve
IT: Information theory
LIC: Limit information curve
MI: Mutual information
PI-RADS: Prostate imaging reporting and data system
PREV: Prevalence of disease
ROC: Receiver operating characteristic
SE: Sensitivity
SP: Specificity
